# Identification of poor prognostic joint locations in an early rheumatoid arthritis cohort at risk of rapidly progressing disease: a post-hoc analysis of the Phase III AGREE study

**DOI:** 10.1186/s41927-022-00252-4

**Published:** 2022-04-14

**Authors:** Patrick Durez, Rene Westhovens, Femke Baeke, Yedid Elbez, Sofie Robert, Harris A. Ahmad

**Affiliations:** 1 Institut de Recherche Expérimentale Et Clinique (IREC), Cliniques Universitaires Saint-Luc, Université Catholique de Louvain, Service de rhumatologie, 1200 Bruxelles, Belgium; 2 Department of Development and Regeneration, Skeletal Biology and Engineering Research Center, KU Leuven, Leuven, Belgium; 3 Bristol-Myers Squibb, Braine L’Alleud, Belgium; 4 Deepscover, Puteaux, France; 5 Bristol-Myers Squibb, Princeton, USA

**Keywords:** Early RA, Abatacept, Joint location, Prognosis, Clinical practice

## Abstract

**Background:**

Rheumatoid arthritis (RA) is a heterogeneous disease with established poor prognostic factors such as seropositivity, joint damage, and high disease activity at an early, treatment-naïve stage of disease. However, few studies have examined if specific joint locations are correlated with these factors in such a population. This analysis explored the potential correlation of individual swollen and erosive joints with other disease characteristics at baseline and with remission rates in a post-hoc analysis of the Phase III randomized AGREE study.

**Methods:**

Methotrexate (MTX)-naïve, erosive, RF- and/or ACPA-positive early RA patients (N = 509) were retrospectively evaluated. Baseline joint swelling was analyzed for large and small joints. Baseline erosions were analyzed for wrist, MCP1–5, IP1, PIP2–5 and MTP1–5. Remission rates were assessed after 6 months of treatment with abatacept (ABA) + MTX (N = 256) or MTX (N = 253). The following statistical tests were used: Chi-Square or Fisher’s exact test (categorical variables); Student’s t-test or Wilcoxon rank-sum test (continuous variables); continuity-corrected Chi-square test (efficacy remission endpoints).

**Results:**

Baseline swelling was most frequent in wrist (91.9%) and MCP2 joint (89.1%), while baseline erosion was most frequent in MTP5 joint (43.5%). Swollen shoulder was significantly correlated (*p* < 0.0001) with swelling of almost all other large or medium joints. Baseline swelling in the knee, temporomandibular joint (TMJ), wrist and elbow was highly correlated (*p* < 0.001) with higher tender and swollen joint counts, higher DAS28(CRP) and higher SDAI and CDAI. Baseline swelling was not correlated with erosion per joint, except for MCP2. The largest difference in mean Boolean remission rates at 6 months was in patients with baseline swollen wrist favoring ABA + MTX (14.0% vs 4.4%; *p* < 0.001).

**Conclusions:**

Swelling in the large and medium joints (knee, TMJ, elbow and wrist) was highly correlated with severe disease activity while MCP2 swelling seemed to be correlated with joint damage. The correlation of joint locations at an early, treatment-naïve stage with poor prognostic factors, higher disease activity and joint damage, could establish a rapidly progressing anatomical pattern in early RA.

*Trial registration*: ClinicalTrials.gov NCT00122382, registered July 2005.

**Supplementary Information:**

The online version contains supplementary material available at 10.1186/s41927-022-00252-4.

## Introduction

Rheumatoid arthritis (RA) is the most frequent inflammatory arthritis worldwide affecting 0.2% to 1.0% of the population [1, 2]. While this autoimmune disease is in general more common in women than men, there might be differences in the pathogenesis of RA according to genetic and environmental factors [3–5]. RA is characterized by symmetrical involvement of synovial joints (mainly small joint of the hands and feet), swelling (hyperplasia) and inflammation (synovitis and osteitis) which may lead to joint destruction, deformity and irreversible disability [6–8]. Early diagnosis and treatment are critical and the current treatment goal in early RA is early and persistent disease remission, prevention of long‐term structural damage and restoration of the patient’s quality of life [9, 10].

Swelling and tenderness of joints have been shown to correlate with functional impairment because of inflammation mainly in early disease but also later on due to consequent joint damage [11–14]. RA patients often present with different areas of joint involvement but limited data exist to identify which specific joint locations may be related to disease severity at an accelerated pace [9]. Long-term radiological follow-up data from the BeSt study revealed association of swelling and tenderness in individual large joints during the first 2 years of treatment with joint damage after 8 years in the same joints. Moreover, large-joint damage, but not small-joint damage, was associated with functional disability [14]. However, in a 12-year prospective cohort of RA patients, for small joints, the association between joint damage and functional ability has been shown to increase with time [11]. Furthermore, other studies showed that the clinical signs of swelling were also associated with later joint damage in large joints, as previously reported for small joints [15, 16]. These data highlight the importance of considering RA not only as a disease of small joints.

Most patients with early stage of disease have swelling in the hands and feet, while swelling of the large joints (shoulders, elbows, hips, knees and ankles) is less prevalent in early RA or could occur later in the disease course [17]. The location of affected joints i.e. the wrist is also critical for patients as it is associated with different functional impairment and impact on quality of life [18].

Prognostic factors are currently considered for establishing the treatment course and prognosis in RA [19]. Three most relevant and most frequently used factors for the prognosis of RA have been identified, i.e. high disease activity including high number of swollen joints, positivity for rheumatoid factor (RF) and/or anti-citrullinated protein-peptide antibodies (ACPA), and the early presence of structural damage; these prognostic factors are incorporated in current treatment recommendations for the management of RA [20]. Of note, in clinical practice, certain prognostic factors or their combinations, do not always serve useful in predicting future joint damage [21]. Treatment response, especially remission, observed at 6 months is associated with long-term benefit [22]. Although to our knowledge joint location has not been assessed, it could be evaluated as a potential measure of prognosis, considering its simplicity and practicality.

Thus, an analysis of existing datasets of early RA patients with poor prognostic factors for joint locations that may be indicative of greater or accelerated disease activity would be of value. Such analysis was conducted for the ‘Abatacept study to Gauge Remission and joint damage progression in methotrexate (MTX)-naïve patients with Early Erosive rheumatoid arthritis (AGREE)’ [23, 24]. Patients in this study had early RA (≤ 2 years) and represented a particularly poor prognostic population, i.e. presence of erosions and seropositivity for RF and/or ACPA, which are associated with poor radiographic, functional and clinical outcomes [25–27].

The current post-hoc analysis of the AGREE study explored whether involvement of specific joint(s) can serve as a poor prognostic factor in RA patients warranting a more intensive treatment approach.

## Methods

### Study objectives

The objectives of this analysis were to: (1) investigate the distribution of the affected joints by assessment of the swelling and erosive joint status in the early seropositive RA cohort of the AGREE study; (2) investigate the cross-sectional correlation of the affected joints with different baseline characteristics and (3) evaluate the remission rates of patients at 6 months by baseline swollen and erosive joint status.

### Study design and population

This post-hoc analysis was based on data from the previously published AGREE Phase III study (ClinicalTrials.gov identifier NCT00122382), in which patients were randomized (1:1) to receive ABA + MTX or MTX alone over a 12-month, double-blind period [24].

Patients eligible for the study were 18 years or older, had RA for 2 years or less, at least 12 tender and 10 swollen joints, C-reactive protein (CRP) 0.45 mg/dl or higher, RF and/or ACPA seropositivity and radiographic evidence of bone erosion of the hands, wrists or feet. Patients were either MTX-naïve or had previous MTX-exposure of 10 mg/week or less for a maximum of 3 weeks, with no MTX administered for 3 months before providing informed consent.

### Data collection and analysis

Prevalence of baseline individual swollen joint status (present, absent) was assessed through a physical examination by the physician for 5 large (elbows, shoulders, temporomandibular joint [TMJ], knees, ankles), 1 medium (wrist) and 15 small joint locations (metacarpophalangeal [MCP] joints 1–5, interphalangeal [IP] joint 1, proximal interphalangeal [PIP] joints 2–5 [together considered as the hand] and metatarsophalangeal [MTP] joints 1–5 [the foot]). Swelling at baseline meant that at least one of the 2 joints (left or right) were swollen. So, each patient had a binary status with/without swollen joint at each site. Swelling resolution was identified as swelling being present at baseline and absent after 6 months of treatment, as assessed through a physical examination by a trained physician. To limit variability, joint assessments were done according to the EULAR handbook and an individual training session performed by an international renowned specialist was foreseen before start of the AGREE study.

Radiographs of the hands and feet were acquired on each patient and sent to a central reading facility for quality control where they were standardized to ensure sufficient image quality for the evaluation of radiographic RA progression. The film-screen system was standardized to ensure sufficient resolution for the evaluation of erosions and joint space narrowing. Prevalence of baseline erosive joint status was assessed by two independent radiologists blinded to treatment arm and chronological sequence for 14 locations in each hand and wrist (PIP2-5 joints, the IP joint of the thumb and 5 MCP joints, the carpometacarpal joint of the thumb, the scaphoid bone, the distal radius and the distal ulna) and 6 joints in each foot (5 MTP joints and the IP joint of digit I, i.e. the great toe), using the total Genant-modified Sharp score (TGSS) [28]. Briefly, both readers were trained and experienced in the scoring of RA by the TGSS as well as certified for the study through the evaluation of a set of test cases and evaluation of agreement between the readers. For the erosion score the locations were scored individually using an eight-point scale from 0 to 3.5 based on the amount of articular bone eroded.

Of note, erosion of the wrist was noted as absence or presence, whatever the 4 sub-joint location. Functional disability was evaluated using the Health Assessment Questionnaire without Disability Index (HAQ-DI), with the HAQ remission cut-off of ≤ 0.5.

Remission rates were assessed according to the DAS28-CRP criteria, Boolean definitions, Clinical Disease Activity Index (CDAI) and Simplified Disease Activity Index (SDAI) as previously described [23].

For each swollen joint location, the difference in clinical response rate (measured as Boolean remission at 6 months) between treatment groups (ABA + MTX vs MTX) was assessed. The 6 months timepoint of assessment was chosen based on findings from a previous retrospective study in patients with RA where treatment response, especially remission, observed at 6 months has been shown to be associated with long-term benefit [22].

### Statistical analysis

Baseline characteristics and demographics were reported using descriptive statistics.

Effect of baseline swollen and erosive joint status on baseline disease characteristics was analyzed with p-values comparing swollen versus non-swollen and erosive versus non-erosive joint status for each parameter. Categorical variables were assessed by Chi-Square test if n ≥ 5 and Fisher’s exact test otherwise. Continuous variables were assessed by Student’s t-test if assumption of normality held or n > 30 and Wilcoxon rank sum test otherwise.

Pairwise association of joints was evaluated with a Chi-square test (significance level of 5%).

The difference in efficacy remission endpoints at 6 months between baseline swollen versus non-swollen and erosive versus non-erosive joint status was tested with a p-value based on a continuity-corrected Chi-square test.

## Results

### Baseline demographics and clinical characteristics

This post-hoc analysis included all 509 patients from the AGREE study [24]. The patients’ demographic and baseline clinical characteristics are described in Table 1 with an overall mean disease duration of 6.5 months and a high disease activity evidenced by an overall mean Disease Activity Score-28 with CRP (DAS28-CRP) of 6.3. As shown in Table 1, the patients exhibited high disease activity, ACPA- and RF-seropositivity, and joint damage in an early MTX-naïve stage.

**Table 1:** Summary of baseline demographics, disease and clinical characteristics of the AGREE study patients (N = 509)

	ABA + MTX (N = 256)		MTX (N = 253)		Total (N = 509)	
	n	Value	n	Value	n	Value
Age in years, mean (SD)	256	50.1 (12.4)	253	49.7 (13.0)	509	49.9 (12.7)
Gender, n (%) female	256	196 (76.6)	253	199 (78.7)	509	395 (77.6)
Race, n (%) White	256	202 (78.9)	253	219 (86.6)	509	421 (82.7)
Disease duration in months, mean (SD)	256	6.2 (7.5)	253	6.7 (7.1)	509	6.5 (7.3)
Tender joints, mean (SD)*	256	31.3 (14.8)	253	30.8 (14.0)	509	31.0 (14.4)
Swollen joints, mean (SD)*	256	22.9 (11.3)	253	21.9 (10.1)	509	22.4 (10.8)
DAS28-CRP, mean (SD)	256	6.3 (1.0)	252	6.2 (1.0)	508	6.3 (1.0)
HAQ DI, mean (SD)	254	1.7 (0.7)	251	1.7 (0.7)	505	1.7 (0.7)
ACPA-positive, n (%)	256	236 (92.2)	253	217 (85.8)	509	453 (89.0)
RF-positive, n (%)	256	246 (96.1)	253	245 (96.8)	509	491 (96.5)
Total x-ray score, mean (SD)	253	7.5 (9.7)	253	6.7 (8.8)	506	7.1 (9.2)
Erosion score, mean (SD)	253	5.4 (6.1)	253	4.8 (5.4)	506	5.1 (5.8)
Joint-space narrowing score, mean (SD)	253	2.1 (4.2)	253	1.9 (4.0)	506	2.0 (4.1)

N, total number of patients; n, number of patients included in each treatment group; SD, standard deviation, ABA, abatacept; MTX, methotrexate; DAS28-CRP, disease activity score in 28 joints-C-reactive protein; HAQ DI, health assessment questionnaire disability index; ACPA, anti-citrullinated protein antibody; RF, rheumatoid factor; Total x-ray score, total Sharp score using the Genant-modified Sharp method

*A total of N = 68 and N = 66 joints were assessed for tender and swollen joints, respectively

### Prevalence of swollen and erosive joint locations

At baseline, swelling was most prevalent in the wrist (91.9%) and in MCP2 (89.1%) and MCP3 (83.6%) joints, while baseline erosion was mostly observed in the wrist (42.5%), the feet, particularly in MTP5 (43.5%), and in MCP1, 2 and 3 (27.6%, 19.5% and 18.6% respectively) (Table 2).

**Table 2:** Prevalence (%) at baseline of swollen, erosive and swollen erosive joints in the total AGREE cohort (N = 509)

	Swelling	Erosion	Swelling and erosion
*Large joint*			
Ankle	79.2	–	
Knee	68.4	–	
Elbow	48.1	–	
Shoulder	33.8	–	
TMJ	8.8	–	
*Medium joint*			
Wrist	91.9	42.5	39.5
*Small joint*			
MCP1	75.1	27.6	22.1
MCP2	89.1	19.5	18.6
MCP3	83.6	18.6	17.4
MCP4	49.8	5.9	3.6
MCP5	50.4	9.1	6.3
IP1	53.6	6.7	3.8
PIP2	74.3	6.9	4.7
PIP3	78.2	11.6	9.3
PIP4	61.0	6.7	5.1
PIP5	51.2	6.1	4.7
MTP1	44.6	24.3	10.1
MTP2	51.8	19.7	12.3
MTP3	51.0	25.0	14.0
MTP4	40.1	17.3	8.5
MTP5	30.0	43.5	12.5

N, number of patients; TMJ, temporomandibular joint; MCP metacarpophalangeal joints; IP, interphalangeal joint; PIP, proximal interphalangeal joints; MTP, metatarsophalangeal joints

### Correlation of individual joint swelling at baseline with disease activity and prognosis

Swollen shoulder was significantly associated with swelling of almost all other large or medium joint locations, while swollen wrist was the least associated joint (Additional file 1).

Baseline disease characteristics that were most highly correlated with swelling of the large or medium joints are shown in Additional file 2; among these, baseline swelling in the knee, TMJ, wrist and elbow was highly correlated with higher tender and swollen joint counts, higher DAS28-CRP and higher SDAI and CDAI.

In terms of function, swelling in the knee and IP1 joint of the hand was the most correlated with a higher HAQ-DI score in swollen versus non-swollen joints (mean [standard deviation]) at baseline (1.8 [0.6] vs 1.5 [0.7], *p* < 0.001 and 1.8 [0.6] vs 1.6 [0.7] *p* < 0.001, respectively).

### Correlation of erosive status of individual small joints at baseline with disease activity and prognosis

When looking at erosion positivity in individual joints and TGSS, baseline swelling did not seem to be correlated with erosive status, only for the MCP2 joint a trend was observed (in swollen and non-swollen MCP2 joints, erosion positivity was 20.8% and 7.3% and mean [standard deviation] TGSS was 7.4 [9.5] and 4.6 [5.5], respectively; Additional file 3).

Men seemed to be more prone to erosions than women; among patients with erosive joints, the prevalence of men was higher than among patients with non-erosive joints for all assessed joints except the wrist and PIP2. Statistically significant differences were observed for MCP1, 2, and 3, PIP3 and 5 and MTP2 (Additional file 4).

ACPA-positivity was also correlated with baseline erosion in the joints of the feet, especially MTP3 (erosive: 98.6%, non-erosive: 87.2%; *p* < 0.01), while there were no differences in the prevalence of RF-positive among patients with or without baseline erosions (data not shown).

### Response to treatment for specific joint locations

As previously described, baseline characteristics of the patients treated with MTX or combined therapy (ABA + MTX) were comparable [24].

In general, combination therapy resulted in higher swelling resolution compared to MTX alone, especially in hand (ABA + MTX: 42.7% vs MTX: 27.9%; *p* < 0.001), wrist (61.2% vs 48.7%; *p* < 0.01) and ankle (57.8% vs 45.3%; *p* < 0.01) but not for the TMJ (84.6% vs 94.7%; *p* < 0.001) (Additional file 5; panel A); similarly, although not significant, association of swelling resolution of individual joints with HAQ-DI remission seemed higher in patients on combination therapy, except for TMJ (Additional file 5; panel B).

**Fig. 1:**
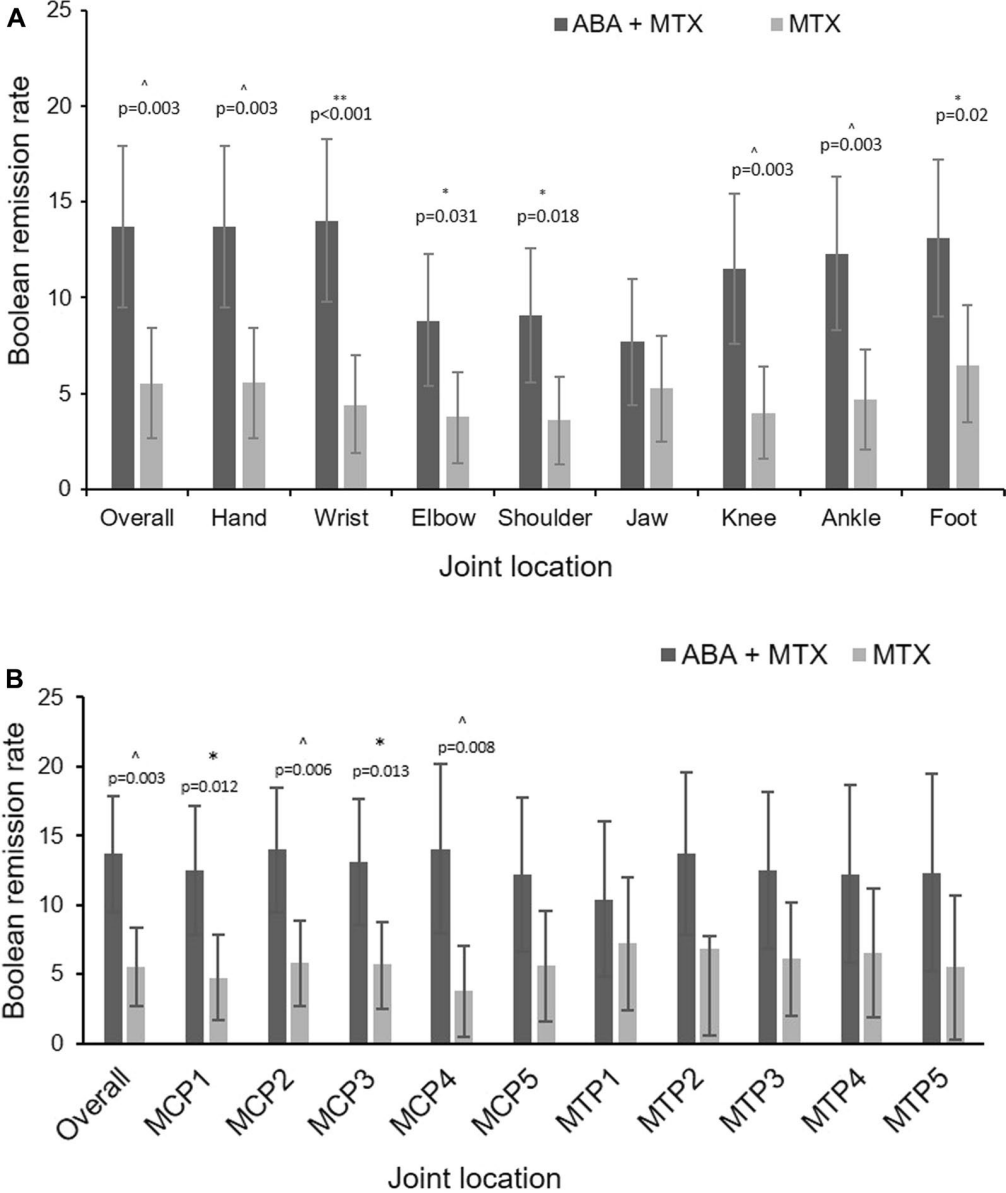
Treatment group comparisons of Boolean remission rates (6 months) in patients with baseline swollen joint status by **A** large and **B** small joint location. ABA, abatacept; MTX, methotrexate; MCP metacarpophalangeal joints; MTP, metatarsophalangeal joints. **p < 0.001; ^*p* < 0.01; *p < 0.05. *p*-value is based on a continuity-corrected chi-square test. The error bars represent 95% confidence interval

Overall, mean Boolean remission rates were higher in patients who received the combination therapy compared to MTX (ABA + MTX: 13.7% vs MTX 5.5%; *p* < 0.01) with the most pronounced difference in the patients with baseline swollen wrist (ABA + MTX: 14.0% vs MTX 4.4%; *p* < 0.001); remission rates were generally lower in patients with baseline swollen elbow, TMJ and shoulder compared to other assessed joints (Fig. 1A).

Boolean remission rates were higher for all assessed small joints in patients receiving combined therapy, with most pronounced differences observed in MCP2 (ABA + MTX: 14.0% vs MTX 5.8%; *p* < 0.01); and MCP4 (ABA + MTX: 14.0% vs MTX 3.8%; *p* < 0.01) (Fig. 1B).

## Discussion

In this cohort of early, seropositive erosive RA patients, about 90.0% of the patients had baseline swelling in the wrist and MCP2 joint, whereas baseline erosion was most frequently observed in the MTP5 joint (43.5%). Swelling in the large and medium joints (i.e. knee, TMJ, elbow or wrist) seems highly correlated with severe disease activity at baseline while baseline MCP2 swelling seems to be correlated with joint damage. Combined therapy with ABA + MTX results in higher remission rates compared to MTX alone, particularly when baseline wrist swelling is present.

Assessment of the joint location by the physician or trained health care professionals appears to be very practical in the evaluation of early RA, the disease course and prognosis, as it is a non-expensive and simple examination (no laboratory tests involved) and there is little variability on YES/NO involvement of a joint comparing to the degree of involvement, as opposed to laboratory tests or evaluation of the disease index which might have inter- and intra-measurement variability. However, although joint count is considered the most specific quantitative measure to assess and monitor the RA status, it is a time-consuming activity which might be problematic for the physicians in their daily practice, and it is not free of the inter- and intra-observer variability [29–31]. Besides its possible role as prognostic or therapeutic biomarker, the affected joint location is also of importance as it will differently affect the patients in their daily activities; from the patient’s perspective, specific joint locations like wrists and feet may be more critical, as swelling, tenderness and pain in hands and feet may affect even simple daily tasks.

In general, wrists, MCP, and PIP joints are the most frequently affected joints in RA patients [13, 32]. In this poor prognostic MTX-naïve RA cohort, this analysis confirms the common joint patterns of RA described in literature, i.e. the high prevalence of baseline MCP2 swelling and baseline MTP5 joint erosions [9, 32–34]. In addition, swollen shoulder status was associated with swollen status of almost all other large joint locations, whereas swollen status of the wrist was the least associated with swollen status of other joints. Validation of clinical markers are useful to determine the best possible therapeutic strategy. Identification of specific joints as negative prognostic factor may facilitate the clinician to select a more intensive therapy.

An association between joint swelling and disease activity in RA has been previously shown in several studies. Similarly as in this analysis, a previous Japanese study in patients with early RA showed that among the affected joints (i.e. joints with either tenderness or swelling), baseline presence of a swollen wrist and knee were associated with high disease activity (as measured using the DAS28-ESR) [9]. A recent prospective, observational study in patients with RA assessed the association between patient-reported flares of swollen and tender joints, increases in disease activity after a clinical examination, and swelling detected by ultrasonography [35]. For patients reporting flares in the hand (either swollen wrist, MCP or PIP), swelling of the affected joint was confirmed by clinical examination and disease activity increased compared with baseline. This study also underlines the importance of patient-reported joint assessment which may help the physicians monitoring the disease progression between the routine visits.

Although it has been previously reported that joint swelling is associated with joint damage in patients with RA [12, 14–16], in this analysis, swelling at baseline did not seem to be related to erosions at baseline (not for presence of erosion in the same joint and total erosion score), except for the MCP2 joint. Whereas various studies evaluated the relation between baseline joint swelling and the development of joint damage on the longer run [12, 14–16], our data linking MCP2 swelling at baseline with baseline erosions might further highlight the importance of the MCP2 swelling as a predominant joint location in early RA to be recognized by all physicians, especially the general practitioners. In a previous study with RA patients, MCP2 and 3 were the most frequently swollen and had the highest erosion scores of all the MCP joints; although no direct relationship between swelling and erosion in these joints has been shown, it was suggested that the mechanisms leading to clinical inflammation and future joint damage might be related [36]. The literature confirms an additional value of imaging techniques such as ultrasonography and magnetic resonance imaging to better evaluate the synovitis in a diversity of joints (also large joints) for predicting flares [37]. However, the main objective in early RA is to achieve clinical remission or at least low disease activity defined by clinical items and not imaging findings. This was confirmed in the ARCTIC and Taser study in which imaging does not add a scientific evaluation in the follow-up of early RA [38, 39]. Additionally, a careful interpretation of imaging by experts is needed since overinterpretation is possible. Overall, men seemed more prone to develop erosions than women (except for the wrist). The results of other studies assessing the association of gender and erosions in RA seem inconclusive, as some studies reported a higher prevalence of erosions in women than in men [40, 41], and other reported erosive disease more prevalent in men [42], or similar in men and women [43, 44].

We also observed that ACPA-positivity was linked with erosion, especially in the joints of the feet, while RF-positivity had no effect on the erosion status. Of note, in this study, ACPA- and/or RF-positivity was an inclusion criterium, so this difference is of interest as it is observed in an already selected patient population. No conclusion could be drawn for an ACPA negative population in our study. Nevertheless, these findings are in line with previous studies reporting that ACPA-positive RA patients have more swollen joints and more joint destruction compared to ACPA-negative patients [36]. A direct link between ACPA and erosions, even in the absence of inflammation/swelling, has been previously reported [45, 46]. Another study showed that ACPA alone, without inflammation, was not associated with erosion, while the combined presence of ACPA and inflammation was, suggesting that joint inflammation functions as key mediator in the development of erosions in ACPA-positive RA [47]. Furthermore, in that study, the erosive joint status was associated with the concomitant presence of ACPA and RF, rather than ACPA alone, suggesting that presence of ACPA alone is not the main and/or single pathogenic factor contributing to joint erosions.

Finally, the mean Boolean remission rates were higher in patients on ABA + MTX compared to MTX alone with the most significant impact of the combined treatment (*p* < 0.001) observed in patients with a swollen wrist at baseline. This observed efficacy is in line with findings of other studies assessing treatment responses to ABA + MTX in RA patients [48, 49]. Compared to MTX alone, treatment with ABA + MTX also resulted in overall better swelling resolution of the large and medium joints. This was particularly evident for the wrist, but not for the TMJ, which could be due to the low number of patients for whom the TMJ was assessed or to the difficulties in the assessment of the swelling of this latter joint by the physician. Moreover, large geographical differences in joint assessments have previously been reported in the Measurement of Efficacy of Treatment in the Era of Outcome in Rheumatology (METEOR) study [50].

The strengths of this study include the purity and the disease severity of the patient cohort included in the analysis, i.e. early RA, RF and/or ACPA seropositivity and erosions which is a rare combination of these three parameters. Joints correlated with these characteristics could be considered as poor prognostic in early, MTX-naïve RA. The limitations of this study include the nature of the analysis, i.e. post-hoc, multicentric, descriptive analysis, and the difficulty of assessing shoulders and TMJ by the physicians, highlighting the need for a careful and adequate examination of these joints, possibly with the use of ultrasound. Furthermore, this analysis included a highly poor prognostic population of patients due to the inclusion criteria, while such population might not reflect the patient population seen in daily clinical practice (e.g. patients seronegative for ACPA and/or RF). It should be also noted that some joints are not frequently swollen in patients with early RA which might make correlation evaluations less reliable. Finally, many individual tests were performed in the current analysis, with no additional measures taken to reduce the risk of multiple testing.

## Conclusions

In this cohort of early, erosive, MTX-naïve RA patients, baseline swelling in the knee, TMJ, wrist and elbow seemed to be correlated with high initial disease activity (i.e. less disease control), whereas baseline MCP2 swelling seemed to be correlated with the greatest degree of early structural joint damage. Outcomes in such patients were more favorable with combined therapy (ABA + MTX) compared to MTX alone, especially when baseline wrist swelling was present. Identification of these poor prognostic joint locations in early RA could indicate a need for more intensive therapy, but further prospective studies in even larger populations with different subset of early RA are still needed.

## Supplementary Information

**Additional file 1:** Supplementary material.

## Data Availability

The data that support the findings of this study are available from Bristol-Myers Squibb but restrictions apply to the availability of these data, which were used under license for the current study, and so are not publicly available. Data are however available from the authors upon reasonable request and with permission of Bristol-Myers Squibb.

## Abbreviations

ABA: Abatacept
ACPA: Anti-citrullinated protein-peptide antibodies
AGRRE: Abatacept study to Gauge Remission and joint damage progression in methotrexate (MTX)-naïve patients with Early Erosive rheumatoid arthritis
CDAI: Clinical Disease Activity Index
CRP: C-reactive protein
DAS28-CRP: Disease Activity Score-28 with CRP
HAQ: Health Assessment Questionnaire
HAQ-DI: Health Assessment Questionnaire without Disability Index
IP: Interphalangeal joint
MCP: Metacarpophalangeal joints
METEOR: Measurement of Efficacy of Treatment in the Era of Outcome in Rheumatology
MTP: Metatarsophalangeal joints
MTX: Methotrexate
N or n: Number
PIP: Proximal interphalangeal joints
RA: Rheumatoid arthritis
RF: Rheumatoid factor
SD: Standard deviation
SDAI: Simplified Disease Activity Index
TGSS: Total Genant-modified Sharp score
TMJ: Temporomandibular joint
