# Supplementary material for: Identification of poor prognostic joint locations in an early rheumatoid arthritis cohort at risk of rapidly progressing disease: a post-hoc analysis of the Phase III AGREE study
Source: BMC Rheumatol. 2022 Apr 14;6:24. doi: 10.1186/s41927-022-00252-4 (PMC9009012; doi:10.1186/s41927-022-00252-4)
Supplement: Supplementary file 1 — Additional file 1: Supplementary material. [file 41927_2022_252_MOESM1_ESM.docx]

**Supplementary material**

**Identification of poor prognostic joint locations in an early rheumatoid arthritis cohort at risk of rapidly progressing disease: a *post-hoc* analysis of the Phase III AGREE study**

Patrick Durez, Rene Westhovens, Femke Baeke, Yedid Elbez, Sofie Robert, Harris A. Ahmad

**Additional file 1.** Pairwise association of baseline swollen joint status (Chi-Square tests; N=509)

|  | **Elbow** | **Foot** | **TMJ** | **Knee** | **Shoulder** | **Wrist** |
| --- | --- | --- | --- | --- | --- | --- |
| **Ankle** | 0.0022^ | 0.0216* | 0.0046^ | <0.0001** | 0.0712 | 0.0028^ |
| **Elbow** |  | 0.0825 | 0.0004** | <0.0001** | <0.0001** | 0.1227 |
| **Foot** |  |  | 0.0063^ | 0.0619 | <0.0001** | 0.4777 |
| **TMJ** |  |  |  | 0.0019^ | <0.0001** | 0.8295 |
| **Knee** |  |  |  |  | <0.0001** | 0.0016^ |
| **Shoulder** |  |  |  |  |  | 0.6933 |
| N, number of patients; TMJ, temporomandibular joint  ******p <0.001, ^p <0.01, *p <0.05 | | | | | | |

**Additional file 2.** Baseline disease characteristics for baseline swollen versus non-swollen knee, TMJ, elbow or wrist joints

|  | **Knee** | | | |  | **TMJ** | | | |  | **Elbow** | | | |  | **Wrist** | | | |
| --- | --- | --- | --- | --- | --- | --- | --- | --- | --- | --- | --- | --- | --- | --- | --- | --- | --- | --- | --- |
|  | **SJC** | | **No SJC** | |  | **SJC** | | **No SJC** | |  | **SJC** | | **No SJC** | |  | **SJC** | | **No SJC** | |
|  | **N** | **value** | **N** | **value** |  | **N** | **value** | **N** | **value** |  | **N** | **value** | **N** | **value** |  | **N** | **value** | **N** | **value** |
| **Tender joints, mean (SD)** | 348 | 33.1 (14.9)** | 161 | 26.5 (12.2) |  | 45 | 40.0 (15.1)** | 464 | 30.1 (14.1) |  | 245 | 34.1 (15.0)** | 264 | 28.1 (13.3) |  | 468 | 31.4 (14.6)* | 41 | 26.3 (12.0) |
| **Swollen joints, mean (SD)** | 348 | 24.8 (11.2)** | 161 | 17.2 (7.3) |  | 45 | 35.9 (13.3)** | 464 | 21.1 (9.5) |  | 245 | 25.3 (11.2)** | 264 | 19.8 (9.6) |  | 468 | 23.0 (10.7)** | 41 | 15.5 (9.4) |
| **DAS28 (CRP), mean (SD)** | 348 | 6.5 (0.9)** | 160 | 5.8 (1.0) |  | 45 | 6.7 (1.0)^ | 463 | 6.2 (1.0) |  | 245 | 6.5 (1.0)** | 263 | 6.0 (0.9) |  | 467 | 6.3 (1.0)** | 41 | 5.6 (1.0) |
| **SDAI, mean (SD)** | 348 | 51.7 (14.4)** | 159 | 41.2 (12.6) |  | 45 | 57.4 (16.1)** | 462 | 47.6 (14.3) |  | 245 | 52.6 (14.8)** | 262 | 44.5 (13.5) |  | 466 | 49.4 (14.5)** | 41 | 37.8 (12.1) |
| **CDAI, mean (SD)** | 348 | 47.8 (12.9)** | 159 | 39.2 (12.4) |  | 45 | 53.3 (14.3)** | 462 | 44.3 (13.0) |  | 245 | 48.8 (13.0)** | 262 | 41.6 (12.7) |  | 466 | 45.9 (13.1)** | 41 | 35.3 (11.5) |
| **HAQ DI, mean (SD)** | 346 | 1.8 (0.6) | 159 | 1.5 (0.7) |  | 45 | 1.9 (0.5) | 460 | 1.7 (0.7) |  | 243 | 1.8 (0.6) | 262 | 1.6 (0.7) |  | 464 | 1.7 (0.7) | 41 | 1.7 (0.7) |
| **ACPA-positive, n (%)** | 348 | 308 (88.5) | 161 | 145 (90.1) |  | 45 | 41 (91.1) | 464 | 412 (88.8) |  | 245 | 211 (86.1) | 264 | 242 (91.7) |  | 468 | 419 (89.5) | 41 | 34 (82.9) |
| **RF-positive, n (%)** | 348 | 337 (96.8) | 161 | 154 (95.7) |  | 45 | 45 (100.0) | 464 | 446 (96.1) |  | 245 | 237 (96.7) | 264 | 254 (96.2) |  | 468 | 451 (96.4) | 41 | 40 (97.6) |
| **Total x-ray score, mean (SD)** | 346 | 7.5 (9.7) | 160 | 6.2 (8.1) |  | 45 | 9.4 (10.1) | 461 | 6.9 (9.1) |  | 243 | 7.1 (8.6) | 263 | 7.1 (9.8) |  | 466 | 7.1 (9.2) | 40 | 7.7 (9.1) |
| **Erosion score, mean (SD)** | 346 | 5.3 (6.0) | 160 | 4.6 (5.2) |  | 45 | 6.0 (6.1) | 461 | 5.0 (5.8) |  | 243 | 5.0 (5.4) | 263 | 5.2 (6.2) |  | 466 | 5.1 (5.8) | 40 | 5.0 (5.3) |
| **Joint-space narrowing score, mean (SD)** | 346 | 2.2 (4.4) | 160 | 1.6 (3.5) |  | 45 | 3.3 (4.4) | 461 | 1.9 (4.1) |  | 243 | 2.1 (4.0) | 263 | 1.9 (4.2) |  | 466 | 2.0 (4.1) | 40 | 2.7 (4.7) |
| N, number of patients included in the analysis; SJC, swollen joint count; n (%), number (percentage) of patients within a category; SD, standard deviation; DAS28, disease activity score in 28 joints; CRP, C-reactive protein; SDAI, Simplified Disease Activity Index; CDAI, Clinical Disease Activity Index; HAQ DI, health assessment questionnaire disability index; ACPA, anti-citrullinated protein antibody; RF, rheumatoid factor; Total x-ray score, total Sharp score using the Genant-modified Sharp method; TMJ, temporomandibular joint.  **p <0.001, ^p <0.01, *p <0.05. | | | | | | | | | | | | | | | | | | | |

**Additional file 3.** Proportion of patients with erosion positivity at baseline (A) and mean TGSS (B) in patients with baseline swollen and non-swollen joints by individual joint location

**A.**

**B.**

MCP metacarpophalangeal joints; IP1H, interphalangeal joint of the thumb; PIP, proximal interphalangeal joints; MTP, metatarsophalangeal joints; TGSS, total Genant-modified Sharp score.

Additional file 4. Prevalence (%) of men and women in erosive and non-erosive joints by swollen joint location at baseline

| Swollen joint | Prevalence male (%) in erosive joints at BL | Prevalence male (%) in non-erosive joints at BL | Prevalence female (%) in erosive joints at BL | Prevalence female (%) in non-erosive joints at BL | P-value |
| --- | --- | --- | --- | --- | --- |
| Wrist | 19.5% | 24.4% | 80.5% | 75.6% | 0.2053 |
| MCP1 | 33.0% | 17.9% | 67.0% | 82.1% | 0.0013^ |
| MCP2 | 33.0% | 20.4% | 67.0% | 79.6% | 0.0103* |
| MCP3 | 36.4% | 19.4% | 63.6% | 80.6% | 0.0008** |
| MCP4 | 38.9% | 22.6% | 61.1% | 77.4% | 0.1163 |
| MCP5 | 31.3% | 25.6% | 68.8% | 74.4% | 0.4941 |
| IP1 | 36.8% | 20.2% | 63.2% | 79.8% | 0.0888 |
| PIP2 | 20.8% | 23.0% | 79.2% | 77.0% | 0.8058 |
| PIP3 | 42.6% | 19.0% | 57.4% | 81.0% | 0.0002** |
| PIP4 | 38.5% | 23.8% | 61.5% | 76.2% | 0.0976 |
| PIP5 | 45.8% | 23.4% | 54.2% | 76.6% | 0.0163* |
| MTP1 | 29.4% | 21.8% | 70.6% | 78.2% | 0.2624 |
| MTP2 | 33.9% | 21.5% | 66.1% | 78.5% | 0.0476* |
| MTP3 | 28.2% | 21.9% | 71.8% | 78.1% | 0.2918 |
| MTP4 | 34.9% | 22.5% | 65.1% | 77.5% | 0.0965 |
| MTP5 | 27.0% | 21.3% | 73.0% | 78.7% | 0.4207 |
| MCP metacarpophalangeal joints; IP, interphalangeal joint; PIP, proximal interphalangeal joints; MTP, metatarsophalangeal joints; BL, baseline.  **p<0.001; ^p<0.01; *p<0.05.p-values for statistical comparisons for erosive vs non-erosive joints are based on chi-square test: | | | | | |

**Additional file 5.** Swelling resolution (A) and association of HAQ-DI resolution and swelling resolution (B) for all joints in patients receiving ABA+MTX or MTX alone (N=509)

**A.**

**
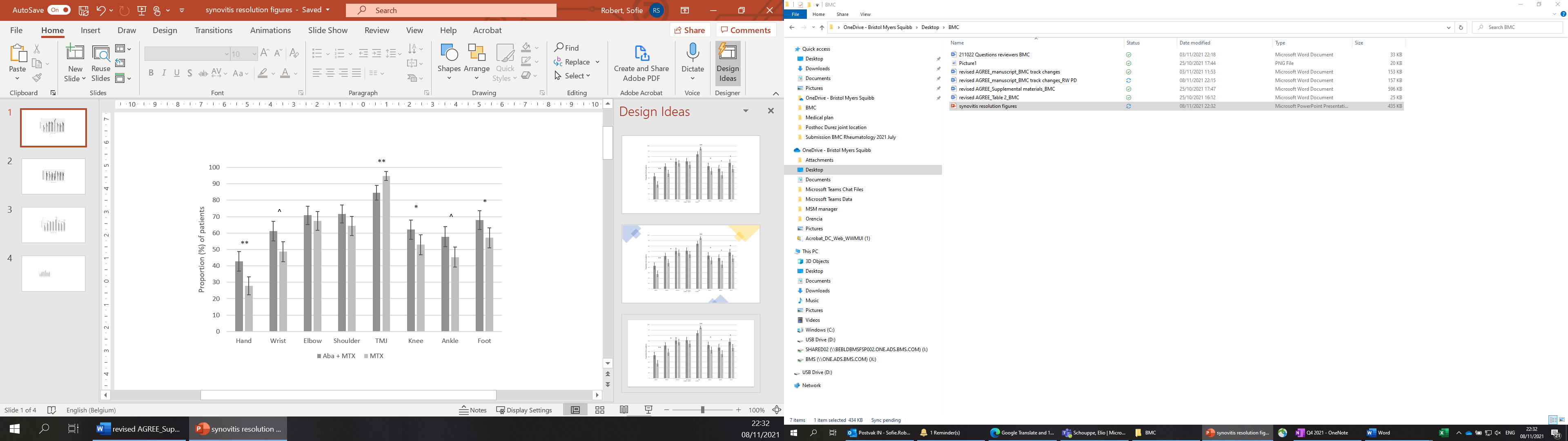
**

**B.**

N, number of patients; ABA, abatacept; MTX, methotrexate; HAQ-DI, health assessment questionnaire-disability index; TMJ, temporomandibular joint.

Fig A and B: ** p <0.001, ^ p <0.01, * p <0.05. Differences in panel B not significant. p-value is based on a continuity corrected chi-square test. The error bars represent 95% confidence interval.
